# Pathogen Proteins Eliciting Antibodies Do Not Share Epitopes with Host Proteins: A Bioinformatics Approach

**DOI:** 10.1371/journal.pone.0000512

**Published:** 2007-06-06

**Authors:** Isaac Amela, Juan Cedano, Enrique Querol

**Affiliations:** Institut de Biotecnologia i de Biomedicina and Departament de Bioquímica i Biologia Molecular, Universitat Autònoma de Barcelona, Barcelona, Spain; University of the Western Cape, South Africa

## Abstract

The best way to prevent diseases caused by pathogens is by the use of vaccines. The advent of genomics enables genome-wide searches of new vaccine candidates, called reverse vaccinology. The most common strategy to apply reverse vaccinology is by designing subunit recombinant vaccines, which usually generate an humoral immune response due to B-cell epitopes in proteins. A major problem for this strategy is the identification of protective immunogenic proteins from the surfome of the pathogen. Epitope mimicry may lead to auto-immune phenomena related to several human diseases. A sequence-based computational analysis has been carried out applying the BLASTP algorithm. Therefore, two huge databases have been created, one with the most complete and current linear B-cell epitopes, and the other one with the surface-protein sequences of the main human respiratory bacterial pathogens. We found that none of the 7353 linear B-cell epitopes analysed shares any sequence identity region with human proteins capable of generating antibodies, and that only 1% of the 2175 exposed proteins analysed contain a stretch of shared sequence with the human proteome. These findings suggest the existence of a mechanism to avoid autoimmunity. We also propose a strategy for corroborating or warning about the viability of a protein linear B-cell epitope as a putative vaccine candidate in a reverse vaccinology study; so, epitopes without any sequence identity with human proteins should be very good vaccine candidates, and the other way around.

## Introduction

Vaccination is the preventive method of choice to fight against microbial pathogens and presents the best cost/benefit ratio among current clinical and pharmaceutical practices. There are many reasons and serious threats which make the development of new advanced vaccines necessary, for example, avian flu the spread of antibiotic-resistant strains of pathogens [1].

The advent of genomics and high-throughput cloning/expression of large sets of genomic ORFs from pathogens makes genome-wide searches of new vaccine candidates possible. This systematic identification of potential antigens and virulence factors of a pathogen without the need for its cultivation has been termed “reverse vaccinology” [2], [3]. The objective is to find proteins eliciting antibodies capable of binding to the bacterial surface, and through interaction with the complement system kill certain pathogen microorganisms. However, current studies show that only a small fraction of the pathogen proteins, most surface-exposed or secreted, appears to elicit antibodies with bactericidal activity [2], [3]. It is generally considered that a bactericidal assay, that is, an antigen that elicits murine antibodies capable of triggering bacterial-cell death *in vitro* in a complement-dependent manner, is a good candidate for human vaccine development [4]–[7]. However, a major obstacle to reverse vaccinology, besides sequence and antigenic variability, is the difficulty to identify, from the pathogen proteome, those proteins that will generate a protective response. Typically, only a very small fraction of the antibodies raised in large-scale antigen-screening studies are bactericidal [3], [8], [9].

Epitope mimicry appears when a stretch of shared sequence, called “mimetope”, exists between a protein of a certain pathogen and a protein of its host. In some cases this may lead to auto-immune phenomena most of them related to several diseases. Different pathogens presenting epitope mimicry could cause auto-immune diseases like, for instance, *Borrelia burgdorferi*, leading to Lyme disease or neuroborreliosis; several Streptococci related to Rheumatic Fever, *Tripanosoma cruzi*, causing Chaga's Disease: *Campylobacter jejuni*, causing Guillain- Barré Syndrome; a group of viruses and also *Chlamydia pneumoniae*, related to Multiple Sclerosis, B3 coxsakieviruses, leading to myocarditis; B4 coxsakieviruses or cytomegaloviruses, leading to Type I Diabetes; HSV-1, causing herpetic stromal kerartitis or some kind of auto-immunity caused by mimicry of Chlamydia pneumoniae outer-membrane proteins and myosin [10]. For several of these kinds of auto-immune diseases, we do not yet know what the possible causal agent is like, for example, with Primary Biliary Cirrhosis, Psoriasis, Scleroderma, Sjögren's Syndrome or Lupus.

In this context, a method to quickly identify potential protective antigens from the pathogen proteome would be very helpful. Our initial hypothesis is that, given that one of the most important tasks of the immune system is the differentiation between self and non-self proteins, this system will discard eliciting antibodies against pathogen proteins sharing epitopes with host proteins. On the other hand, epitope mimicry could cause several auto-immune diseases as stated above [10]. Moreover, nowadays it is thought that humoral response against foreign proteins can distinguish between dangerous proteins and nondangerous ones [11]. For this reason we have focused our study only on pathogen proteins, probably dangerous, and not on human self-microbiota proteins, most of them nondangerous.

A sequence-based computational analysis has been carried out to exemplify what has been said above. We studied the complete set of exposed surface proteins –the surfome- of most of the sequenced respiratory human-pathogen microorganisms with the aim of elucidating the presence of stretches of a shared sequence, which have characteristics of putative T-dependent B-cell epitopes, between human and pathogen proteins. This group of bacterial proteins, recently labelled the surfome [7], is susceptible to generating antibody immune response via B-cell epitopes, and it has been reported that some of them effectively deal with this type of response. The paradigm analysed here could be used to describe how the bacterial proteins sharing linear B-cell epitopes with human proteins avoid immunoreactivity against the host if these sequence stretches tend to produce antibodies. Many questions like the following ones were formulated: Are these produced antibodies non-protective ones? Or are these proteins not producing antibody responses even though they were able to do it? Auto-immune diseases should appear if the exposed proteins of the bacterial pathogens share linear B-cell epitopes with any human protein, so this study may be useful to identify them [2], [3]. These findings may also help us to identify proteins which should not be used as putative vaccine candidates in a reverse vaccinology study. Moreover, the existence of a mechanism to avoid auto-immunity could be proposed. On the other hand, we therefore studied the current, already-known linear B-cell epitopes to elucidate if these sequence stretches either share or do not share common regions with human proteins. This study may help us to say that novel epitope mimicries could generate new auto-immune diseases and could reinforce the hypothesis of the existence of the mechanism to avoid auto-immunity proposed above.

A linear or continuous B-cell epitope is a specific region of an antigen to which an antibody binds and its constitutive residues are sequential in the primary sequence of the protein. In contrast, conformational or discontinuous B-cell epitopes are highly conformational-dependant and its constitutive residues are non-sequential in the primary sequence. That is why conformational B-cell epitopes were not included in our field of study, thus we restricted our study to the group of linear B-cell epitopes. The lack of many databases for conformational B-cell epitopes and the poorly developed method for predicting it from structure, enforced this decision. We have to take into account that only the protein sequences sharing certain sequence identity regions were considered if these two proteins are different and do not have anything in common in their annotation. This decision was assumed due to the fact that there exist many proteins of completely different organisms grouped in the same family or being considered as protein-like ones and obviously these proteins are condemned to share common regions, so they were not included in our field of study. The antibodies, in fact, recognise a small part of a big molecule. In this sense, two quite different proteins could be considered that would be recognised by the same antibody if both shared a small epitope of five to six amino acids, more or less, which is the usually accepted minimal length for a linear B-cell epitope.

As a brief comment of our results, we found that none of the well-known protective antigens under our study present putative, common linear B-cell epitopes with the host proteins, which demonstrates the existence of a system that tries to avoid autoimmunity that select linear B-cell epitopes not sharing sequence stretches with human proteins. On the other hand, we found several pathogen proteins which share sequence stretches with the host ones, so we recommend not to using these proteins as putative vaccine candidates in a reverse vaccinology study. These proteins are a small group of the total surfome studied and they also elicit antibodies with great difficulty, suggesting the same immunotolerance effect that tries to avoid autoimmunity mentioned here [12].

## Materials and Methods

### Generation of the Datasets

With the aim of proposing a method applicable in a previous reverse vaccinology study, databases were made of the exposed proteins from the up-to-date, sequenced main human bacterial respiratory pathogens, which are: *Neisseria meningitidis serogroup B, Legionella pneumophila* (Lens strain), *Streptococcus pneumoniae, Haemophilus influenzae, Pseudomonas aeruginosa, Streptococcus pyogenes serotype M1, Yersinia pestis, Bordetella bronchiseptica, Staphylococcus aureus* (COL strain), *Pasteurella multocida, Bordetella parapertussis, Bordetella pertussis, Chlamydia pneumoniae (Chlamydophila pneumoniae)* and *Mycoplasma pneumoniae*. Besides, to attempt to demonstrate our previous proposal that some selectivity exists to try to avoid the auto-immune response, we obtained a complete set of linear B-cell epitopes from the three most complete and current epitope databases.

On one hand, a surface-protein sequence database for each pathogen was created downloading the protein sequences from the HAMAP tool under the ExPASy web server [13], [14]. Furthermore, exhaustive searches in the NCBI [www.ncbi.nlm.nih.gov/entrez], SCIRUS for scientific information [www.scirus.com] and in specialised journals helped us to find proteins with certain interest for our study because of their reported capacity to generate antibody immune response via B-cell epitope activation. These protein sequences were added to each pathogen database. A total of 2175 proteins sequences from the surfome [7] of the studied pathogens was analysed.

On the other hand, we developed different computational programmes to obtain the human pathogen section of the Bcipep database [15], the most complete and specific B-cell epitope database now available from which 2275 pathogen linear B-cell epitopes were obtained. In addition, we performed a similar procedure to collect the B-cell epitope portion of the IEDB database [16], an extensive immune epitope database appeared in early-2006 with a total of 2154 linear B-cell epitopes. Finally, we could computationally retrieve all the linear B-cell epitopes from the AntiJen database [17], an important immunological database that contains quantitative binding data for epitope peptides from which we could obtain 2924 entries. Thus a total of 7353 linear B-cell epitopes has been studied and most of them were formed by at least six or more amino acids.

### Procedure

These database files, 14 consisting of the different pathogen-exposed proteins and 3 of the most powerful recent databases, including linear B-cell epitopes, were generated to analyse if there exist sequence identities between human proteins and the collected surface microorganism proteins or between the same human proteins and the complete set of linear B-cell epitopes obtained. This study may lead us to request a method based on sequence similarity, like the one obtained here, that ought to be applied in a previous step of a reverse vaccinology study. Furthermore, this should help us to discover putative pathogens enhancing auto-immunity after their infection or demonstrating that some selectivity exists to avoid the auto-immune response.

For each of the pathogens analysed, downloading was started from the HAMAP tool under the ExPASy web server of all protein sequences whose annotation included the following keywords: Outer, membrane, lipoprotein, adhesin, surface, secreted or exposed. Then, as said above, extensive searches were carried out through the NCBI web server, the SCIRUS web site and several specialised journals, with the aim of finding scientific articles talking about proteins that generate antibody immune response via B-cell epitope activation or could produce auto-immunity and probably act as putative vaccine candidates. An initial sequence database (ID) was made as explained before. Additionally, this initial database file for each pathogen was used to run a BLASTP algorithm analysis [18] against the protein non-redundant database at the NCBI ftp site [www.ncbi.nlm.nih.gov/Ftp]. If we found that some of these proteins had stretches of shared sequence with human proteins, thanks to this preliminary BLASTP analysis, we constructed a more extensive sequence database (ED) for each pathogen using, once again, the HAMAP tool under the ExPASy web server, now including all protein sequences that contain, in their annotation, the hypothetical, probable, conserved or putative protein annotation statements also considering the above-described keywords. This ED file of protein sequences for each pathogen served as input for a more accurate BLASTP analysis.

The BLASTP output file for each pathogen analysed had been carefully scrutinised to obtain a last file (LF) including the alignments that comprise local, significant sequence similarity between the pathogen query protein and a subject human one if one exists. A stretch of shared sequence was considered as a putative mimetope if there were at least 5 sequential, equal amino acids or if it is a region to take into account because of its amino acid physico-chemical properties similarity. For the LF file of exposed protein databases of the 14 pathogens analysed, we checked to see if these similar stretches correspond to transmembrane regions because a B-cell epitope cannot exist in a transmembrane region. This was done by applying TransMem, a programme for predicting transmembrane domains in proteins [19]. We also checked to see if these stretches of shared sequence coincide with the signal peptide section of the protein, when these are close to the N-terminal extreme, using the SignalP 3.0 Server [20]. Lastly, it was checked to determine if these stretches are predicted as putative linear B-cell epitopes using prediction servers like ANTIGENIC [21], ABCpred [22], and BcePred [23].

### B-Cell Epitope Database

Thanks to the Bcipep, IEDB and AntiJen databases, we could group together a total of 7353 linear B-cell epitopes, preparing them for the subsequent BLASTP analysis against the human protein, non-redundant database at the NCBI ftp site.

### Exposed Protein Databases

Regarding *Neisseria meningitidis serogroup B*, the ID contained 62 protein sequences [5], [9], [24]–[28] and the ED was made up of 263 protein sequences. In the case of *Legionella pneumophila* (Lens strain), the ID had 17 protein sequences [29]–[31] and the ED consisted of 352 protein sequences. The *Streptococcus pneumoniae* study allowed us to obtain an ID that comprised 27 protein sequences [7], [32]–[36] and an ED of 338 protein sequences. Regarding *Haemophilus influenzae*, we were able to generate an ID of 25 protein sequences [37]–[41]. In this case, we did not search for more protein sequences because we did not find any previous sequence-identity region between the proteins from the ID and human ones, so the database remained consisting of a total of 25 protein sequences. Concerning *Pseudomonas aeruginosa*, we were able to assemble an ID of 34 protein sequences [42]–[49] and a huge ED that contained 569 protein sequences. The *Streptococcus pyogenes serotype M1* study allowed us to obtain an ID of 30 protein sequences [7], [50]–[58] and an ED of 278 protein sequences. Regarding *Yersinia pestis*, we only created an ID of 32 protein sequences [59]–[61]. We did not increase this ID because we did not find any region with a sequence identity between the proteins from this database and human proteins in the preliminary BLASTP analysis. Concerning *Bordetella bronchiseptica*, we could group together 46 protein sequences in its ID [62]–[64]. Nothing else was added to this ID because we did not find stretches of shared sequence between the proteins from this database and human ones. In the case of *Staphylococcus aureus (strain COL)*, we were able to generate an ID of 43 protein sequences [65]–[67] and we could extend it to 490 protein sequences, producing a large ED. Regarding *Pasteurella multocida* assembled an ID of 20 protein sequences [68]. Nothing else was added to this ID because we did not find any local sequence-identity regions between the sequences from this database and human proteins. We made the same analysis for the rest of the pathogens in our study, which are *Bordetella parapertussis, Bordetella pertussis, Chlamydia pneumoniae (Chlamydophila pneumoniae)* and *Mycoplasma pneumoniae*. In the cases of *Bordetella parapertussis* and *Bordetella pertussis,* we were able to generate an ID of 28 and 37 sequences respectively [69]–[73]. For *Chlamydia pneumoniae (Chlamydophila pneumoniae)*, the ID was made up of 68 protein sequences [74]–[77]. Regarding *Mycoplasma pneumoniae*, the ID contained 198 protein sequences. These last four pathogens have not got any sequence-identity region between the proteins from the IDs and human ones, so the databases were not enlarged. Taking into account all of the pathogens analysed, we were able to study the sequence of a total of 2175 proteins.

## Results and Discussion

### B-Cell Epitope Analysis

In terms of our study, after analysing the three database files of linear B-cell epitopes obtained via BLASTP [18], an exhaustive review of the output files obtained led us to the proposal that some selectivity really does exist to try to avoid auto-immunity. We concluded this because we could see that none of those 7353 linear B-cell epitopes shared any sequence identity region with human proteins capable of generating antibodies despite the already known epitopes that generate auto-antibodies and cause auto-immune diseases, the allergies caused by some epitopes, or certain sequence identities found between some artificial peptides and human proteins after their administration. We found that well-known protective antigens do not present putative common linear B-cell epitopes with the host proteins, so this fact reinforces what we have postulated before that some selectivity may exist to try to avoid auto-immunity. Moreover, pathogen proteins which share sequence stretches with the host ones elicit antibodies with great difficulty, demonstrating the immunotolerance effect [12].

### Exposed Protein Analysis

The LF for each pathogen analysed, coming from the output file after the BLASTP analysis [18], which contains the alignments comprising local, significant sequence similarity between the pathogen query protein and a subject human one, have been carefully scrutinised. Despite the huge amount of exposed proteins and pathogens analysed, around 2000, we could only consider approximately 20 protein alignments, representing only 1% of the total of proteins analysed. Consequently, we can say that, due to the probable existing selectivity mechanism to avoid auto-immunity already mentioned, the finding of pathogen-exposed proteins that have a stretch of shared sequence, considered as a putative linear B-cell epitope, with human proteins is very difficult. As said before, epitope mimicry between proteins may lead to auto-immune phenomena most of them related to several diseases, but also pathogen proteins which share sequence stretches with the host ones elicit antibodies with great difficulty demonstrating the immunotolerance effect. The way of trying to avoid this mimicry and the presence, in some cases, of this immunotolerance effect, is to reinforce the difficulty in finding regions of sequence identity between pathogen and host proteins.

Here we show an example of a procedure, applied in this case only to the main human, bacterial respiratory pathogens, which we think might be genuinely helpful for the development of new vaccines when corroborating or advising the viability of a pathogen surfome protein [7] as a putative vaccine candidate in a reverse vaccinology study [2], [3].

As stated above, only 20 proteins of a total of 2000 analysed share a significant sequence-identity region with human proteins, so we strongly advise against recommending these proteins as putative vaccine candidates. On the contrary, the rest of the proteins should be good vaccine candidates. Although this situation has to be considered, we show below some of the alignments between the exposed-pathogen proteins and several human proteins that we think would be more significant to highlight as an example of a previous massive analysis in a reverse vaccinology study.

#### *Streptococcus pneumoniae* and *Chlamydia pneumoniae*:

**Figure 1:**
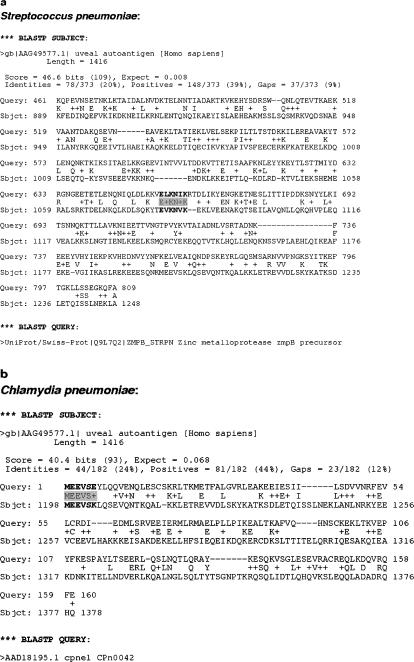
Example of mimetope identification. (a) Partial sequence alignment coming from the output file of the BLASTP algorithm analysis between the exposed-protein database from *Streptococcus pneumoniae* and the non-redundant protein database. A putative linear B-cell epitope is highlighted. (b) Partial sequence alignment coming from the output file of the BLASTP algorithm analysis between the exposed-protein database from *Chlamydia pneumoniae* and the non-redundant protein database. A putative linear B-cell epitope is highlighted.

After the BLASTP output file analysis of these two pathogens, we could see that there exist several bacterial proteins sharing a significant sequence-identity region with human proteins, but we found a very interesting particular case on which we focused in more detail (Figures 1a, 1b). Two proteins of these pathogens have a stretch of shared sequence with a human protein that is considered to have a uveal auto-antigen [78]. Futhermore, the region of each of the two proteins is not included in the predicted transmembrane sections. These regions also are not part of a signal peptide and there are also certain references highlighting that these proteins may elicit antibody immune response [32]–[36], [74]–[77]. These two proteins are the Zinc metalloprotease zmpB precursor in the case of *Streptococcus pneumoniae* and the Cpn0042 protein for *Chlamydia pneumoniae*. Additionally, the stretch of shared sequence for the *Streptococcus pneumoniae* protein does not correspond to the signal peptide because it is not close to the N-terminal extreme, and the stretch of shared sequence for *Chlamydia pneumoniae* protein is not a predicted signal peptide either. Even though the epitope prediction servers already mentioned could not corroborate that these sequence sections correspond to putative linear B-cell epitopes, we considered this case to be an important one.

In our supposed preliminary analysis of a reverse vaccinology study, we should recommend, although these two proteins previously seem to be good putative vaccine candidates, not using these proteins for the development of new vaccines as they probably generate antibodies against the human protein with which they share a stretch of sequence. Moreover, we have to consider that the human protein has an auto-antigen, so we propose that perhaps a previous infection with *Sterptococcus pneumoniae* or *Chlamydia pneumoniae* could promote an auto-immune reaction at the uveal tract apart from producing infection by themselves.

#### *Pseudomonas aeruginosa* and *Chlamydia pneumoniae*:

**Figure 2:**
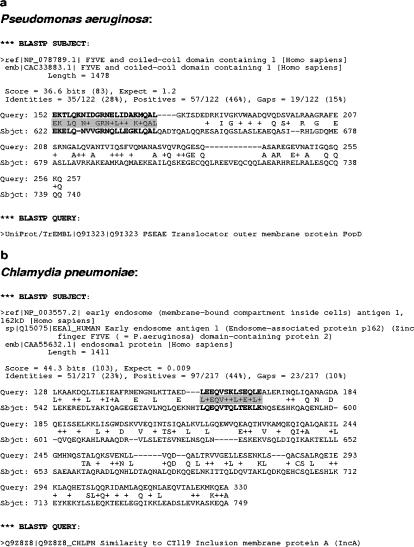
Example of mimetope identification. (a) Partial sequence alignment coming from the output file of the BLASTP algorithm analysis between the exposed-protein database from *Pseudomonas aeruginosa* and the non-redundant protein database. A putative linear B-cell epitope is highlighted. (b) Partial sequence alignment coming from the output file of the BLASTP algorithm analysis between the exposed-protein database from *Chlamydia pneumoniae* and the non-redundant protein database. A putative linear B-cell epitope is highlighted.

The precise analysis of the BLASTP output file for these two pathogens allowed us to identify two proteins containing a significant sequence-identity region with a human protein completely differently annotated from the pathogen one (Figures 2a, 2b). Additionally, these sections do not correspond to transmembrane regions and there are also certain references highlighting the antibody elicitation and therefore the consequential immune response [45]–[49], [74]–[77]. Futhermore, the epitope-prediction servers already mentioned could corroborate that the two identity regions correspond to putative linear B-cell epitopes. These proteins are the Translocator outer membrane protein PopD, a constituent of the *Pseudomonas aeruginosa* Type III Apparatus, and the Inclusion membrane protein A in the case of *Chlamydia pneumoniae*. Obviously, the stretches of shared sequence do not correspond to the signal peptide because they are not close to the N-terminal extreme of the protein.

We considered this example a truly important one for showing why a protein should not be used as a vaccine candidate after a preliminary analysis in a reverse vaccinology study. As is seen in the above alignments, the two pathogen proteins share a significant sequence-identity region with human proteins, so they are candidates for generating an antibody response against the human protein with which they share this stretch of sequence. Moreover, these human proteins are part of the FYVE domain containing proteins and are required for the formation of early-endosomal membranes that are the main resource for phagocyte action [79]–[83]. For this reason, the probable antibody elicitation against these proteins could generate a serious auto-immune problem for the endosome-mediated action in the human defence system.

#### *Streptococcus pyogenes*:

**Figure 3:**
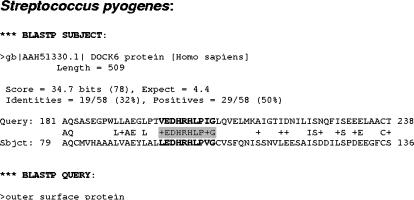
Example of mimetope identification. Partial sequence alignment coming from the output file of the BLASTP algorithm analysis between the exposed-protein database from *Streptococcus pyogenes* and the non-redundant protein database. A putative linear B-cell epitope is highlighted.

The final BLASTP output file gave us a remarkable result indicating that there exists only one pathogen protein sharing a certain sequence identity-region with a human protein differently annotated from the pathogen one (Figure 3). In addition to this protein having all the characteristics to be a putative vaccine candidate and to elicit antibody immune response [55], [56], the epitope-prediction servers already mentioned could corroborate that the sequence-identity region corresponds to a putative linear B-cell epitope and the transmembrane-prediction server shows that this sequence identity region does not coincide with the predicted transmembrane ones. Obviously, the stretches of shared sequence do not correspond to the signal peptide because they are not close to the N-terminal extreme of the protein.

As can be seen here, this pathogen protein has a stretch of shared sequence with a human protein called DOCK6. Consequently, the putative antibodies generated against this exposed-pathogen protein could attack DOCK6, leading to auto-immune effects. DOCK6 promotes neurite outgrowth [84], so this protein is necessary for neural development and we believe that a lack of DOCK6 could be quite dangerous for human health. Therefore, we also recommend not considering this outer-surface protein as a putative vaccine candidate.

### Final Considerations

We have proposed a protein sequence analysis that may be applied before a reverse vaccinology study for which we have shown several examples. Our proposal may be used in this kind of massive analysis for the development of new vaccines when corroborating or warning about the viability of a linear B-cell epitope as a putative vaccine candidate. Therefore, epitopes without any sequence identity with human proteins should be very good vaccine candidates, and the other way round.
